# Estimation of Anti-HIV Activity of HEPT Analogues Using MLR, ANN, and SVM Techniques

**DOI:** 10.1155/2013/795621

**Published:** 2013-12-30

**Authors:** Basheerulla Shaik, Tabassum Zafar, Vijay K. Agrawal

**Affiliations:** ^1^Department of Applied Sciences, National Institute of Technical Teachers' Training & Research, Bhopal, Madhya Pradesh 462002, India; ^2^CSIR-Advanced Materials and Processes Research Institute (AMPRI), Bhopal, Madhya Pradesh 462064, India

## Abstract

The present study deals with the estimation of the anti-HIV activity (log1/*C*) of a large set of 107 HEPT analogues using molecular descriptors which are responsible for the anti-HIV activity. The study has been undertaken by three techniques MLR, ANN, and SVM. The MLR model fits the train set with *R* ^2^=0.856 while in ANN and SVM with higher values of *R* ^2^ = 0.850, 0.874, respectively. SVM model shows improvement to estimate the anti-HIV activity of trained data, while in test set ANN have higher *R* ^2^ value than those of MLR and SVM techniques. *R*_*m*_^2^ = metrics and ridge regression analysis indicated that the proposed four-variable model MATS5e, RDF080u, T(O*⋯*O), and MATS5m as correlating descriptors is the best for estimating the anti-HIV activity (log 1/*C*) present set of compounds.

## 1. Introduction

Undoubtedly Human immunodeficiency virus (HIV) infection is considered to be a deadly disease by the international community including the World Health Organization (WHO), UNAIDS. The WHO in its reports has said that AIDS has killed more than 25 million people since 1981 which is most the destructive pandemics in the history.

It is also a well-known fact that a lentivirus (a member of the retrovirus family) causes *acquired immunodeficiency syndrome* (AIDS) [1, 2], damaging immune system and leading to life-threatening infections. A report published in 2007 reveals that approximately 36 million people suffered due to HIV infection. An estimated 2.1 million people were even killed that year including 330,000 children. Another study also reveals that 2.5 million people developed new infections [3–6]. Unfortunately the number of deaths is still rising due to this deadly disease.

Just to overcome the problem scientists are working day and night and a number of RT inhibitors including various nonnucleoside RT inhibitors (NNRTIs) have been discovered as new anti-HIV agents. They have better blocking potential and have been proved to be effective [7–9]. These compounds 1-[2-Hydroxyethoxy) methyl]-6-(phenylthio)-thymine (HEPT) are known for targeting enzyme allosteric site which are less toxic and found to have more stable than nucleoside RT inhibitors.

Many efforts have been made to model the anti-HIV activity of HEPT derivatives in the past using 2D, 3D, and holographic (HQSAR) methods [10–13]. Quantitative structure activity relationship studies were carried out in order to build models for the estimation of binding affinities (Δ*G*_*b*_) of HEPT and nevirapine analogues with reverse transcriptase [14]. Similarly, Agrawal et al. [15, 16] have successfully reported use of physicochemical as well as topological indices for modeling anti-HIV activities of HEPT analogues.

**Figure 1:**
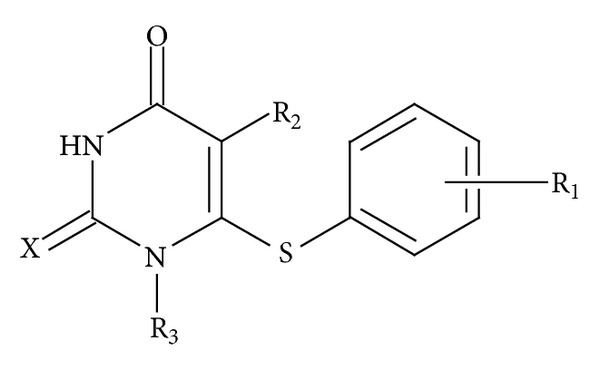
General structure of HEPT compounds used in the present study.

In continuation to these studies we now report modeling of anti-HIV activity of 1-[2-Hydroxyethoxy) methyl]-6-(phenylthio)-thymine (HEPT) derivatives (Figure 1) using graph theoretical descriptors in which distances and connectivity have been considered. The general structure of HEPT compounds used in the present study is demonstrated in Figure 1. The structural details are presented in Table 1. This Table also shows the experimental anti-HIV activity of compounds.

A close look of Figure 1 and the activity data presented in Table 1 indicates that the anti-HIV activity mainly dependent upon the type and number of substituent *R*_1_ in the benzene moiety.

## 2. Materials and Methods

### 2.1. Experimental Data

The structural details as well as anti-HIV activity (log1/*C*) of 107 HEPT analogues are reported in Table 1. The RT inhibition data in terms of log1/*C* have been taken from the literature [12]. All the chemical structures were drawn with the help ACD labs software which helps in the calculation of topological descriptors. These descriptors were calculated using Dragon software using mol file generated by Chem sketch software.

### 2.2. Selection Molecular Descriptors and Training/Test Set for External Validation

In the present study for estimating the anti-HIV activity of 107 HEPT analogues we have used a pool of descriptors classified into 20 different groups. The descriptor selection is carried out by stepwise regression analysis (forward selection method using NCSS ver. 8 [17]. These selected descriptors are recorded in Table S1 (see Supplementary Material available online at http://dx.doi.org/10.1155/2013/795621). The data set was divided into training and test sets using random sampling technique in which 80% (84 compounds) of the data is taken as training set and the remaining 20% (23 compounds) as test set for the MLR, ANN, and SVM analyses.

## 3. Results and Discussion

**Figure 2:**
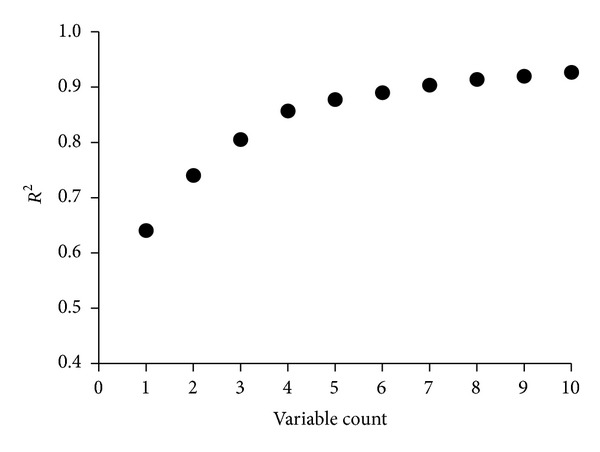
Correlation of variable count against *R* ^2^.

The data (Table 2) was subjected to regression analysis which subsequently gave a correlation matrix showing intercorrelation among the selected descriptors and also with the anti-HIV (log1/*C*) activity. The same has been presented in Table S2. The variable selection for multiple regression analysis has indicated the possibility of using only ten models for modeling the anti-HIV activity (log1/*C*). These models are reported in Table S3. All these models are generated as a result of successive addition of one to ten descriptors. However, correlation of number of descriptors present in the model with *R* ^2^ (Figure 2) indicated that at the most we can use only four to five descriptors for obtaining statistically most significant model. The statistically significant models obtained from MLR analysis are reported below.

### 3.1. MLR Results

#### 3.1.1. One-Variable Model

Successive regression analysis indicated that in one-variable model Moran autocorrelation—lag 5/weighted by atomic Sanderson electronegativites (MATS5e) as correlating descriptor is the best model for modelling the log1/*C*. This model is as follows:

(1) $$\begin{array}{c} \log\frac{1}{C}=-10.9800\left(\pm 0.9083\right)\text{MATS}5\text{e}+4.6503\\ N=84,\;\;\text{Se}=0.8423,\;\;R^{2}=0.6406,\\ F\text{-ratio}=146.132,\;\;Q=0.9501. \end{array}$$

Here and hereafter *N* is number of compounds, Se is standard error, *R* ^2^ is squared correlation coefficient, *R*_*A*_^2^ is adjusted *R* ^2^, *F*-ratio is Fishers ratio, and *Q* is Pogliani quality factor [18–20].

The negative coefficient of MATS5e indicates that the decrease in its magnitude will enhance the activity (log1/*C*).

#### 3.1.2. Two-Variable Model

When (RDF080u) the unweighted radial distribution function 8.0 is added to the above model the model shows significant improvement in all the parameters. The *R* ^2^ value changes from 0.6406 to 0.7402. Similarly improvement in adjusted *R* ^2^ also shows that the addition of (RDF080u) parameter is justified. The improved model is as follows:

(2) log1C=−9.5891(±0.8161)MATS5e +0.0899(±0.0161)RDF080u+3.5920,N=84,  Se=0.7205,  R2=0.7402,RA2=0.7337,  F-ratio=115.364,  Q=1.1940.

The above model indicates that decrease in MATS5e and increase in RDF080u will improve the log1/*C* values.

#### 3.1.3. Three-Variable Model

When T(O ⋯ O), which is a parameter which takes care of distance between O atom, is added to the previously stated two-parametric model a three-parametric model is yielded as below. Here the change in *R* ^2^ and also *Q* value suggests that the model is better than the earlier one:

(3) log1C=−7.9071(±0.7818)MATS5e +0.0979(±0.0141)RDF080u −0.0162(±0.0031)T(O⋯O)+4.1554,N=84,  Se=0.6277,  R2=0.8053,RA2=0.7980,  F-ratio=110.273,  Q=1.4295.

Here the negative coefficient of T(O ⋯ O) indicates that the decrease in topological distance between (O ⋯ O) will favour the exhibition of the anti-HIV activity (log1/*C*).

#### 3.1.4. Four-Variable Model

Addition of Moran auto correlation—lag 5/weighted by atomic masses MATS5 m to the above three-parametric model yielded a four-parametric model. A drastic improvement in variance is observed (*R* ^2^ changes from 0.8053 to 0.8566) The model is given as follows:

(4) log1C=−5.0974(±0.8573)MATS5e +0.0862(±0.0124)RDF080u −0.0150(±0.0027)T(O⋯O) −3.2147(±0.6045)MATS5m+4.2720,N=84,  Se=0.5420,  R2=0.8566,RA2=0.8493,  F-ratio=117.976,  Q=1.7075.

Here the coefficient of MATS5 m is negative. This indicates that lower value of MATS5 m will favour the log1/*C* value for the compounds used in the present study.

A close look at (4) reveals that MATS5e (Moran autocorrelation—lag 5/weighted by atomic Sanderson electronegativites) and MATS5 m (Moran auto correlation—lag 5/weighted by atomic masses) play dominant role in exhibiting the activity. They belong to 2D autocorrelation category. The brief description of the descriptors is given in Table 2.

The predicted log1/*C* values of training set compounds using the above four-parametric model are recorded in Table 1 and plotted against their experimental values. Such a correlation is demonstrated in Figure S1. The above reported model (4) has further been used to predict the log1/*C* values of remaining 23 compounds which are in test set. Such predicted values are also recorded in Table 1. The predicted *R* ^2^ value for the model has been obtained by plotting a graph between observed and estimated log1/*C* values for the compounds and is demonstrated in Figure S1. The *R*_pred_^2^. comes out to be 0.814 confirming that the proposed model is meaningful.

**Figure 3:**
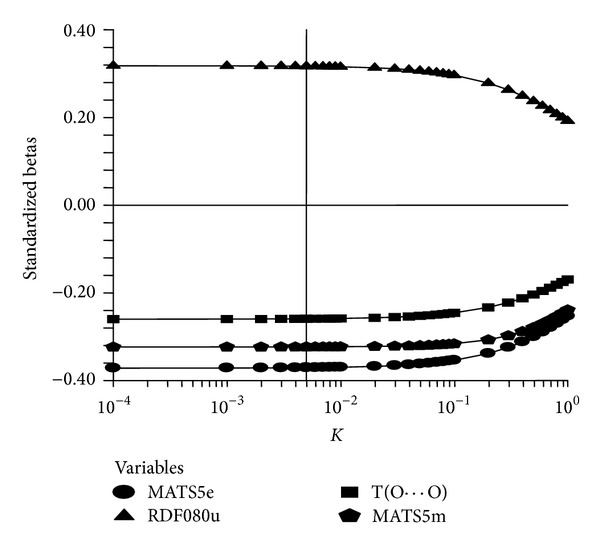
Ridge trace.

**Figure 4:**
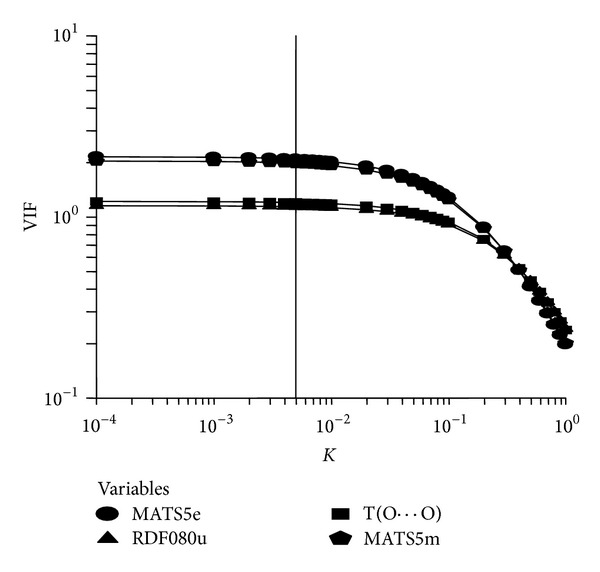
Variance inflation factor.

The above findings confirm that for the estimation of anti-HIV activity (log1/*C*) of present set of compounds a four-variable model containing MATS5e, RDF080u, T(O ⋯ O), and MATS5m as correlating descriptors is the most appropriate model. The Ridge analysis (Table 3) indicates that all the Ridge parameters are well within the allowed values indicating that the proposed model is most suitable and statistically significant. Ridge trace and variance inflation factor for the four variable model were recorded in Figures 3 and 4, respectively.

These four descriptors were further used in artificial neural network (ANN) and support vector machine (SVM) techniques. However the methodology, validation techniques, and model performance evaluation by these two methods is previously discussed by Agrawal et al. [21–23]. The observed and predicted values of log1/*C* of the training as well as the test data using the ANN and SVM techniques are reported in Table S4.

### 3.2. ANN and SVM Results

Artificial neural network (ANN) and support vector machine (SVM) analyses were carried out using STATISTICA Data Miner software Ver. 10 [24]. The initial architecture of the ANN selected was four neurons in the input layer and three neurons in the hidden layer and one output neuron selected by automated network search function. The input neurons correspond to four selected descriptors of the best MLR model. The optimization was done with 10-fold cross-validation. When the entire training data is trained in the network it gives *R* ^2^ = 0.850, RMSE = 2.193, and MAE = 0.24. Using the trained network the test set was used for prediction and gives *R* ^2^ = 0.878, RMSE = 0.823, and MAE = 0.171. A plot of the observed and predicted values of log1/*C* of the training as well as the test data using the ANN model is shown in Figure S2.

The SVM regression type 1 was selected for training the data to obtain capacity *C* and Epsilon (*ε*) and gamma (*γ*) values. In order to find the optimum values of two parameters (*γ* and *ε*), the tenfold cross-validation based on the training set was performed and values giving the lowest RMSE were selected. Using the selected parameters (*γ* = 0.14, *ε* = 0.20, and *C* = 110) final training run was carried out on entire training set resulting in predicted log1/*C* values. The statistical parameters of this model come out to be RMSE = 0.148, *R* ^2^ = 0.874, and MAE = 0.01 for the training set. This SVM model is used to predict log1/*C* values of the test set. The predicted statistical values of SVM model are RMSE = 1.87, *R* ^2^ = 0.867, and MAE = 0.393. A plot of the observed and predicted values of log1/*C* of the training as well as the test set using the SVM model is shown in Figure S3.

The average *r*_*m*_^2^ and Δ*r*_*m*_^2^ were calculated for judging the quality of the proposed model using *r*_*m*_^2^ metric method. It is well established that for an acceptable QSAR model the value of “average *r*_*m*_^2^” should be >0.5 and “Δ*r*_*m*_^2^” should be <0.2 [25, 26]. In our study two different variants of this parameter, *r*_*m*_^2^ and Δ*r*_*m*_^2^, were calculated for both the training (internal validation) and test (external validation) sets in addition to the total dataset (overall validation). The *r*_*m*_^2^, Δ*r*_*m*_^2^ values for all the training, test, and overall data set (MLR, ANN, and SVM) are reported in Table 4.

A close observation of this table clearly indicates that all the values obtained from the *r*_*m*_^2^ metrics are in favour of the four-parametric model proposed by us.

Randomization test is performed to investigate the probability of chance correlation for the best models. Generally in randomization test the dependent variable (log1/*C*) is randomly shuffled and new QSAR models are investigated using the original descriptors. After performing the test, the results indicate that the coefficient of determination obtained by chance is low while the RMSE values are high. This clearly indicates that the models obtained in this study are better than those obtained by chance. The randomization test results are shown in Figure S4.

### 3.3. Comparison with Other QSAR Studies

Luco and coworkers [12] proposed QSAR-based multiple regression analysis and pls methods for anti-HIV activity of 107 HEPT analogues. They developed QSAR-based models on the entire dataset and found that the best model involves 11 correlating descriptors with statistical quality given by *R* ^2^ = 0.9044. It is interesting to compare our results with the results of Luco and coworkers. Our model is with four correlating parameters having the *R* ^2^ = 0.856 in training set and *R*_Pred_^2^ = 0.814 in test case. MLR technique is better than the previously reported one by Luco et al.; in addition to that we have also applied ANN (artificial neural network) and SVM (support vector machine) techniques in which the statistical parameters are better especially with ANN method.

## 4. Conclusions

A comparison of results from the model performance demonstrates that the SVM model predicts the binding affinity of the compounds more accurately than ANN and MLR models for the train dataset. While for test set prediction, ANN model was better. The proposed models could identify and provide some important information which is responsible for anti-HIV activity. These models could be used for designing new HEPT derivatives.

## Supplementary Material

Table S1: Calculated values of the descriptors used in the present study.
Table S2: Correlation matrix.
Table S3: Variable selection for multiple regression analysis.
Table S4: Observed and estimated log 1/C values of eq 4 using ANN and SVM techniques.
Table S5: Values of the r^2^_m_ metrics judging the quality of the model.
Figure S1: Correlation between observed and estimated values of log 1/C using eq. 4.
Figure S2: Correlation between observed and estimated values of log 1/C using eq. 4. (ANN).
Figure S3: Correlation between observed and estimated values of log 1/C using eq. 4. (SVM).

## Figures and Tables

**Table 1:** Structural details of the compounds with their anti-HIV activity (log1/C) values used in the present study.

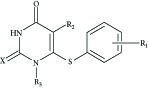							
Comp. No	R_1_	R_2_	R_3_	X	Obs. log 1/C	Est. log 1/C (MLR)	Residual (MLR)

1	2-Me	Me	CH_2_OCH_2_CH_2_OH	O	4.150	4.685	−0.535
2	2-NO_2_	Me	CH_2_OCH_2_CH_2_OH	O	3.850	4.495	−0.645
3	2-OMe	Me	CH_2_OCH_2_CH_2_OH	O	4.720	4.831	−0.111
4	3-Me	Me	CH_2_OCH_2_CH_2_OH	O	5.590	5.520	0.070
5	3-Et	Me	CH_2_OCH_2_CH_2_OH	O	5.570	5.612	−0.042
6	3-t-Bu	Me	CH_2_OCH_2_CH_2_OH	O	4.920	4.891	0.029
7	3-CF_3_	Me	CH_2_OCH_2_CH_2_OH	O	4.350	5.011	−0.661
8	3-F	Me	CH_2_OCH_2_CH_2_OH	O	5.480	5.132	0.348
9	3-Cl	Me	CH_2_OCH_2_CH_2_OH	O	4.890	5.544	−0.654
10	3-Br	Me	CH_2_OCH_2_CH_2_OH	O	5.240	4.821	0.419
11	3-I	Me	CH_2_OCH_2_CH_2_OH	O	5.000	5.037	−0.037
12	3-NO_2_	Me	CH_2_OCH_2_CH_2_OH	O	4.470	3.976	0.494
13	3-OH	Me	CH_2_OCH_2_CH_2_OH	O	4.090	4.882	−0.792
14	3-OMe	Me	CH_2_OCH_2_CH_2_OH	O	4.660	4.507	0.153
15	3,5-Me_2_	Me	CH_2_OCH_2_CH_2_OH	O	6.590	6.792	−0.202
16	3,5-Cl_2_	Me	CH_2_OCH_2_CH_2_OH	O	5.890	5.372	0.518
18	3-COOMe	Me	CH_2_OCH_2_CH_2_OH	O	5.100	4.033	1.067
19	3-COMe	Me	CH_2_OCH_2_CH_2_OH	O	5.140	4.296	0.844
20	3-CN	Me	CH_2_OCH_2_CH_2_OH	O	5.000	4.952	0.048
21	H	CH_2_CH=CH_2_	CH_2_OCH_2_CH_2_OH	O	5.600	5.849	−0.249
22	H	Et	CH_2_OCH_2_CH_2_OH	S	6.960	6.793	0.167
24	H	i-Pr	CH_2_OCH_2_CH_2_OH	S	7.230	7.380	−0.150
25	3,5-Me_2_	Et	CH_2_OCH_2_CH_2_OH	S	8.110	7.691	0.419
27	3,5-Cl_2_	Et	CH_2_OCH_2_CH_2_OH	S	7.370	6.996	0.374
28	H	Et	CH_2_OCH_2_CH_2_OH	O	6.920	6.595	0.325
30	H	i-Pr	CH_2_OCH_2_CH_2_OH	O	7.200	7.982	−0.782
31	3,5-Me_2_	Et	CH_2_OCH_2_CH_2_OH	O	7.890	7.794	0.096
32	3,5-Me_2_	i-Pr	CH_2_OCH_2_CH_2_OH	O	8.570	8.727	−0.157
33	3,5-Cl_2_	Et	CH_2_OCH_2_CH_2_OH	O	7.850	6.822	1.028
35	H	Me	CH_2_OCH_2_CH_2_OH	O	5.150	5.015	0.135
36	H	Me	CH_2_OCH_2_CH_2_OH	S	6.010	5.218	0.792
37	H	I	CH_2_OCH_2_CH_2_OH	O	5.440	5.443	−0.003
41	H	CH=CPh_2_	CH_2_OCH_2_CH_2_OH	O	6.070	6.118	−0.048
42	H	Me	CH_2_OCH_2_CH_2_OMe	O	5.060	5.329	−0.269
44	H	Me	CH_2_OCH_2_CH_2_OCOPh	O	5.120	5.058	0.062
45	H	Me	CH_2_OCH_2_Me	O	6.480	5.020	1.460
46	H	Me	CH_2_OCH_2_CH_2_Cl	O	5.820	5.247	0.573
47	H	Me	CH_2_OCH_2_CH_2_N_3_	O	5.240	5.397	−0.157
48	H	Me	CH_2_OCH_2_CH_2_F	O	5.960	4.919	1.041
49	H	Me	CH_2_OCH_2_CH_2_Me	O	5.480	5.522	−0.042
50	H	Me	CH_2_OCH_2_Ph	O	7.060	6.175	0.885
52	H	Et	CH_2_OCH_2_Me	S	7.580	7.221	0.359
53	3,5-Me_2_	Et	CH_2_OCH_2_Me	O	8.240	8.030	0.210
54	3,5-Me_2_	Et	CH_2_OCH_2_Me	S	8.300	8.293	0.007
56	3,5-Me_2_	Et	CH_2_OCH_2_Ph	O	8.550	8.856	−0.306
59	H	i-Pr	CH_2_OCH_2_Me	O	7.990	7.623	0.367
60	H	i-Pr	CH_2_OCH_2_Ph	O	8.510	8.549	−0.039
61	H	i-Pr	CH_2_OCH_2_Me	S	7.890	7.718	0.172
62	H	i-Pr	CH_2_OCH_2_Ph	S	8.140	8.479	−0.339
63	H	Me	CH_2_OMe	O	5.680	5.341	0.339
64	H	Me	CH_2_OBu	O	5.330	5.405	−0.075
65	H	Me	Et	O	5.660	6.443	−0.783
66	H	Me	Bu	O	5.920	6.171	−0.251
67	3,5-Cl_2_	Et	CH_2_OCH_2_Me	S	7.890	6.913	0.977
68	H	Et	CH_2_O–i-Pr	S	6.660	6.841	−0.181
69	H	Et	CH_2_O–c-Hex	S	5.790	6.395	−0.605
70	H	Et	CH_2_OCH_2_–c-Hex	S	6.450	7.042	−0.592
71	H	Et	CH_2_OCH_2_C_6_H_4_(4-Me)	S	7.110	7.152	−0.042
72	H	Et	CH_2_OCH_2_C_6_H_4_(4-Cl)	S	7.920	7.182	0.738
73	H	Et	CH_2_OCH_2_CH_2_Ph	S	7.040	6.522	0.518
75	H	Et	CH_2_O–i-Pr	O	6.470	6.478	−0.008
76	H	Et	CH_2_O–c-Hex	O	5.400	6.208	−0.808
77	H	Et	CH_2_OCH_2_–c-Hex	O	6.350	7.016	−0.666
78	H	Et	CH_2_OCH_2_CH_2_Ph	O	7.020	6.354	0.666
79	H	c-Pr	CH_2_OCH_2_Me	S	7.020	7.115	−0.095
80	H	c-Pr	CH_2_OCH_2_Me	O	7.000	6.865	0.135
84	4-F	Me	CH_2_OCH_2_CH_2_OH	O	3.600	4.192	−0.592
85	4-Cl	Me	CH_2_OCH_2_CH_2_OH	O	3.600	4.002	−0.402
88	4-OH	Me	CH_2_OCH_2_CH_2_OH	O	3.560	3.526	0.034
92	3-CONH_2_	Me	CH_2_OCH_2_CH_2_OH	O	3.510	4.475	−0.965
93	H	COOMe	CH_2_OCH_2_CH_2_OH	O	5.180	4.652	0.528
94	H	CONHPh	CH_2_OCH_2_CH_2_OH	O	4.740	5.393	−0.653
95	H	SPh	CH_2_OCH_2_CH_2_OH	O	4.680	5.176	−0.496
96	H	CCH	CH_2_OCH_2_CH_2_OH	O	4.740	5.408	−0.668
97	H	CCPh	CH_2_OCH_2_CH_2_OH	O	5.470	5.015	0.455
99	H	COCHMe_2_	CH_2_OCH_2_CH_2_OH	O	4.920	5.579	−0.659
100	H	COPh	CH_2_OCH_2_CH_2_OH	O	4.890	5.085	−0.195
101	H	CCMe	CH_2_OCH_2_CH_2_OH	O	4.720	5.130	−0.410
102	H	F	CH_2_OCH_2_CH_2_OH	O	4.000	3.886	0.114
103	H	Cl	CH_2_OCH_2_CH_2_OH	O	4.520	4.214	0.306
104	H	Br	CH_2_OCH_2_CH_2_OH	O	4.700	4.460	0.240
105	H	Me	CH_2_OCH_2_CH_2_OCH_2_Ph	O	4.700	5.157	−0.457
106	H	Me	H	O	3.600	4.575	−0.975
107	H	Me	Me	O	3.820	4.535	−0.715

*Test *							
17	3,5-Me_2_	Me	CH_2_OCH_2_CH_2_OH	S	6.660	7.005	−0.345
23	H	Pr	CH_2_OCH_2_CH_2_OH	S	5.000	6.644	−1.644
26	3,5-Me_2_	i-Pr	CH_2_OCH_2_CH_2_OH	S	8.300	8.499	−0.199
29	H	Pr	CH_2_OCH_2_CH_2_OH	O	5.470	6.532	−1.062
34	4-Me	Me	CH_2_OCH_2_CH_2_OH	O	3.660	4.382	−0.722
38	H	CH=CH_2_	CH_2_OCH_2_CH_2_OH	O	5.690	6.366	−0.676
39	H	CH=CHPh	CH_2_OCH_2_CH_2_OH	O	5.220	5.726	−0.506
40	H	CH_2_Ph	CH_2_OCH_2_CH_2_OH	O	4.370	5.183	−0.813
43	H	Me	CH_2_OCH_2_CH_2_OAc	O	5.170	4.385	0.785
51	H	Et	CH_2_OCH_2_Me	O	7.720	7.080	0.640
55	H	Et	CH_2_OCH_2_Ph	O	8.230	7.271	0.959
57	H	Et	CH_2_OCH_2_Ph	S	8.090	7.184	0.906
58	3,5-Me_2_	Et	CH_2_OCH_2_Ph	S	8.140	8.940	−0.800
74	3,5-Cl_2_	Et	CH_2_OCH_2_Me	O	8.130	6.881	1.249
81	H	Me	CH_2_OCH_2_CH_2_OC_5_H_11_	O	4.460	4.538	−0.078
82	2-Cl	Me	CH_2_OCH_2_CH_2_OH	O	3.890	4.738	−0.848
83	3-CH_2_OH	Me	CH_2_OCH_2_CH_2_OH	O	3.530	4.726	−1.196
86	4-NO_2_	Me	CH_2_OCH_2_CH_2_OH	O	3.720	3.835	−0.115
87	4-CN	Me	CH_2_OCH_2_CH_2_OH	O	3.600	4.794	−1.194
89	4-OMe	Me	CH_2_OCH_2_CH_2_OH	O	3.600	3.588	0.012
90	4-COMe	Me	CH_2_OCH_2_CH_2_OH	O	3.960	4.208	−0.248
91	4-COOH	Me	CH_2_OCH_2_CH_2_OH	O	3.450	3.765	−0.315
98	3-NH_2_	Me	CH_2_OCH_2_CH_2_OH	O	3.600	5.115	−1.515

**Table 2:** Brief description of the descriptors used in the present study.

S. number	Symbol	Descriptor type	Meaning
1	MATS5e	2D autocorrelation	Moran autocorrelation—lag 5/weighted by atomic Sanderson's electronegativities
2	RDF080u	RDF descriptors	Radial distribution function 8.0/unweighted
3	T(O*⋯*O)	Topological descriptors	The sum of topological distance between (O*⋯*O)
4	MATS5m	2D autocorrelation	Moran autocorrelation—lag 5/weighted by atomic masses

**Table 3:** Ridge regression parameters for (4).

Model number	Parameters used	VIF	*λ*_*i*_	*T*	*K*
(4)	MATS5e	2.1512	2.1261	0.4649	1.0000
	RDF080u	1.1535	0.9763	0.8670	2.1800
	T(O*⋯*O)	1.2186	0.6042	0.8206	3.5200
	MATS5m	2.0363	0.2932	0.4911	7.2500

VIF: variance inflation factor; *λ*_*i*_: eigenvalue; *T*: tolerance; *K*: condition number.

**Table 4:** Values of the *r* ^2^_*m*_ metrics judging the quality of the model.

S. number	Method	Sets	Average *r* ^2^_*m*_	Δ *r* ^2^_*m*_
1	MLR	Train	0.799	0.114
		Test	0.597	0.170
		Overall	0.732	0.133

2	ANN	Train	0.825	0.079
		Test	0.671	0.133
		Overall	0.799	0.045

3	SVM	Train	0.817	0.102
		Test	0.561	0.182
		Overall	0.731	0.125
