# Supplementary material for: Estimation of Anti-HIV Activity of HEPT Analogues Using MLR, ANN, and SVM Techniques
Source: Int J Med Chem. 2013 Dec 30;2013:795621. doi: 10.1155/2013/795621 (PMC4207402; doi:10.1155/2013/795621)
Supplement: Supplementary file 1 — Table S1: Calculated values of the descriptors used in the present study. Table S2: Correlation matrix. Table S3: Variable selection for multiple regression analysis. Table S4: Observed and estimated log 1/C values of eq 4 using ANN and SVM techniques. Table S5: Values of the r2 m metrics judging the quality of the model. Figure S1: Correlation between observed and estimated values of log 1/C using eq. 4. Figure S2: Correlation between observed and estimated values of log 1/C using eq. 4. (ANN). Figure S3: Correlation between observed and estimated values of log 1/C using eq. 4. (SVM). [file 795621.f1.pdf]

Supplementary material

Table S1 Calculated values of the descriptors used in the present study

Mol ID	MATS5e	RDF080u	T(O..O)	MATS5m
Train	0.009	11.695	33	0.017
Train	-0.107	11.854	103	-0.062
Train	0.005	12.762	64	-0.138
Train	-0.029	13.354	33	-0.138
Train	0.001	19.817	33	-0.041
Train	0.039	18.545	33	0.089
Train	-0.054	14.028	33	0.078
Train	-0.055	10.527	33	-0.052
Train	-0.053	15.24	33	-0.057
Train	-0.049	7.758	33	-0.039
Train	-0.038	11.431	33	-0.025
Train	-0.056	13.25	111	0.019
Train	-0.051	12.748	68	-0.084
Train	-0.013	14.376	68	0.016
Train	-0.051	19.837	33	-0.325
Train	-0.072	11.444	33	-0.075
Test	-0.055	22.52	18	-0.243
Train	-0.032	14.175	111	-0.012
Train	0	14.772	72	0.053
Train	-0.039	10.606	33	-0.019
Train	-0.087	16.199	33	-0.072
Train	-0.182	15.651	18	-0.16
Test	-0.126	19.88	18	-0.089
Train	-0.301	10.755	18	-0.285
Train	-0.184	18.789	18	-0.352
Test	-0.288	18.839	18	-0.437
Train	-0.212	15.63	18	-0.176
Train	-0.167	14.343	33	-0.227
Test	-0.12	19.906	33	-0.133
Train	-0.271	18.953	33	-0.37
Train	-0.164	20.19	33	-0.448
Train	-0.256	21.998	33	-0.544
Train	-0.2	15.545	33	-0.213
Test	0.006	10.909	33	0.095
Train	-0.032	11.34	33	-0.03
Train	-0.027	12.809	18	0.008
Train	-0.072	13.085	33	-0.053
Test	-0.146	14.798	33	-0.177
Test	-0.057	19.347	33	0.003
Test	-0.014	18.761	33	0.088

Train	0	29.506	33	0.063
Train	-0.065	14.902	33	0.02
Test	0.041	14.603	58	0.021
Train	-0.056	12.981	58	-0.078
Train	-0.042	9.22	14	0.016
Train	-0.034	9.456	14	-0.061
Train	-0.033	13.127	14	-0.011
Train	-0.028	7.094	14	-0.032
Train	-0.041	13.538	14	-0.026
Train	-0.057	20.399	14	-0.02
Test	-0.193	16.619	14	-0.187
Train	-0.229	17.059	6	-0.125
Train	-0.196	18.891	14	-0.417
Train	-0.236	21.731	6	-0.322
Test	-0.198	17.72	14	-0.209
Train	-0.201	27.73	14	-0.429
Test	-0.234	15.551	6	-0.146
Test	-0.242	28.36	6	-0.336
Train	-0.309	10.507	14	-0.336
Train	-0.31	20.628	14	-0.351
Train	-0.367	9.879	6	-0.253
Train	-0.369	17.962	6	-0.27
Train	-0.04	9.788	14	-0.072
Train	-0.08	11.558	14	0.019
Train	-0.168	3.532	4	-0.333
Train	-0.112	11.258	4	-0.13
Train	-0.241	11.986	6	-0.146
Train	-0.22	15.493	6	-0.063
Train	-0.196	12.745	6	-0.036
Train	-0.19	20.487	6	-0.039
Train	-0.191	19.993	6	-0.085
Train	-0.216	17.895	6	-0.111
Train	-0.198	12.564	6	-0.077
Test	-0.217	13.123	14	-0.181
Train	-0.184	12.967	14	-0.112
Train	-0.162	12.445	14	-0.077
Train	-0.158	21.911	14	-0.081
Train	-0.166	11.994	14	-0.128
Train	-0.287	10.524	6	-0.175
Train	-0.243	9.042	14	-0.244
Test	-0.026	9.443	33	0.058
Test	-0.015	9.363	33	-0.024
Test	-0.02	14.628	72	-0.053
Train	0.008	8.224	33	0.079

Train	-0.006	7.438	33	0.139
Test	-0.083	10.779	119	0.002
Test	-0.048	9.697	33	0.02
Train	0.011	7.609	72	0.083
Test	0.036	9.764	72	0.082
Test	-0.021	12.793	76	0.042
Test	-0.077	10.543	119	0.008
Train	-0.013	15.928	72	0.049
Train	-0.133	10.334	83	-0.017
Train	-0.176	11.669	58	-0.027
Train	-0.031	15.132	33	0.02
Train	-0.12	7.527	33	-0.115
Train	-0.051	12.987	33	0.044
Test	-0.042	8.859	33	-0.112
Train	-0.122	16.05	58	-0.053
Train	-0.127	13.23	58	0.033
Train	-0.066	11.262	33	-0.014
Train	0.063	4.125	33	-0.023
Train	0.022	5.576	33	-0.021
Train	-0.009	5.859	33	-0.041
Train	-0.06	12.795	33	0.009
Train	-0.028	0.987	4	-0.042
Train	0.017	1.213	4	-0.095

Table S2 Correlation matrix

	log1/C	MATS5e	RDF080u	T(O..O)	MATS5m
log1/C	1				
MATS5e	-0.80673	1			
RDF080u	0.535881	-0.33248	1		
T(O..O)	-0.57943	0.428095	-0.12211	1	
MATS5m	-0.77796	0.730472	-0.3803	0.378901	1

Table S3 Variable selection for multiple regression analysis

Model No	R^2	R^2-Change	Variable names
1	0.6406	0.6406	MATS5e
2	0.7402	0.0996	MATS5e,
3	0.8053	0.0651	MATS5e, RDF080u, T(O..O)
4	0.8566	0.0513	MATS5e, RDF080u, T(O..O) MATS5m
5	0.8775	0.0209	MATS5e, RDF080u, T(O..O), MATS5m, GATS2v
6	0.8897	0.0122	MATS5e, RDF080u, T(O..O), MATS5m, GATS2v, H-050
7	0.9036	0.0140	MATS5e, RDF080u, MATS5m, GATS2v, H-050, nCs, nO
8	0.9138	0.0101	MATS5e, RDF080u, MATS5m, GATS2v, H-050, nCs, Mor28m, nO
9	0.9198	0.0060	MATS5e, RDF080u, MATS5m, GATS2v, H-050, nCs, Mor28m, E2u, nO
10	0.9267	0.0069	MATS5e, MATS7e, RDF080u, MATS5m, GATS2v, nCs, Mor28m, BLTA96, P2m, nO

Table S4 Observed and estimated log 1/C values of eq 4 using ANN and SVM techniques.

Comp . No	Obs. log1/C	Est. Log 1/C ANN	Residual ANN	Est. Log 1/C SVM	Residual SVM
1	4.150	4.644	-0.494	4.729	-0.579
2	3.850	4.121	-0.271	4.734	-0.884
3	4.720	4.845	-0.125	4.861	-0.141
4	5.590	5.628	-0.038	5.594	-0.004
5	5.570	5.599	-0.029	5.607	-0.037
6	4.920	4.840	0.080	4.866	0.054
7	4.350	4.622	-0.272	4.903	-0.553
8	5.480	5.084	0.396	5.112	0.368
9	4.890	5.490	-0.600	5.566	-0.676
10	5.240	4.726	0.514	4.710	0.530
11	5.000	4.966	0.034	5.039	-0.039
12	4.470	3.671	0.799	4.496	-0.026
13	4.090	4.733	-0.643	4.873	-0.783
14	4.660	4.283	0.377	4.561	0.099
15	6.590	6.779	-0.189	6.745	-0.155
16	5.890	5.349	0.541	5.365	0.525
18	5.100	3.781	1.319	4.549	0.551
19	5.140	4.055	1.085	4.394	0.746
20	5.000	4.862	0.138	4.933	0.067
21	5.600	5.736	-0.136	5.823	-0.223
22	6.960	6.884	0.077	6.898	0.062
24	7.230	7.796	-0.566	7.438	-0.208
25	8.110	7.971	0.139	7.753	0.357
27	7.370	7.090	0.280	7.088	0.282
28	6.920	6.753	0.167	6.569	0.351
30	7.200	8.054	-0.854	7.710	-0.510
31	7.890	7.801	0.089	7.491	0.399
32	8.570	8.607	-0.037	8.078	0.492
33	7.850	6.887	0.963	6.762	1.088
35	5.150	4.965	0.185	5.024	0.126
36	6.010	5.132	0.878	5.311	0.699
37	5.440	5.365	0.075	5.443	-0.003
41	6.070	5.709	0.361	5.235	0.835
42	5.060	5.065	-0.005	5.264	-0.204
44	5.120	4.930	0.190	5.021	0.099
45	6.480	4.781	1.699	5.026	1.454
46	5.820	5.193	0.627	5.326	0.494
47	5.240	5.353	-0.113	5.539	-0.299
48	5.960	4.718	1.242	4.870	1.090
49	5.480	5.505	-0.025	5.676	-0.196
50	7.060	6.128	0.932	6.188	0.872
52	7.580	7.161	0.419	7.375	0.205
53	8.240	8.359	-0.119	8.094	0.146
54	8.300	8.444	-0.144	8.393	-0.093
56	8.550	8.509	0.041	8.385	0.165
59	7.990	8.143	-0.153	7.697	0.293
60	8.510	8.604	-0.094	8.460	0.050
61	7.890	7.976	-0.086	7.891	-0.001
62	8.140	8.422	-0.282	8.555	-0.415
63	5.680	5.315	0.365	5.436	0.244
64	5.330	5.153	0.177	5.437	-0.107
65	5.660	6.405	-0.745	6.161	-0.501
66	5.920	6.255	-0.335	6.423	-0.503

67	7.890	6.988	0.902	7.121	0.769
68	6.660	6.617	0.043	6.947	-0.287
69	5.790	6.143	-0.353	6.501	-0.711
70	6.450	6.673	-0.223	6.957	-0.507
71	7.110	6.959	0.151	7.180	-0.070
72	7.920	7.079	0.841	7.305	0.615
73	7.040	6.418	0.622	6.684	0.356
75	6.470	6.490	-0.020	6.599	-0.129
76	5.400	6.139	-0.739	6.304	-0.904
77	6.350	6.755	-0.405	6.872	-0.522
78	7.020	6.429	0.591	6.486	0.534
79	7.020	7.237	-0.217	7.305	-0.285
80	7.000	7.195	-0.195	6.937	0.063
84	3.600	3.973	-0.373	4.110	-0.510
85	3.600	3.565	0.035	3.833	-0.233
88	3.560	3.572	-0.012	3.679	-0.119
92	3.510	4.123	-0.613	4.507	-0.997
93	5.180	4.247	0.933	4.672	0.508
94	4.740	4.913	-0.173	5.219	-0.479
95	4.680	5.015	-0.335	5.162	-0.482
96	4.740	5.416	-0.676	5.264	-0.524
97	5.470	4.742	0.728	4.958	0.512
99	4.920	5.092	-0.172	5.388	-0.468
100	4.890	4.482	0.408	4.912	-0.022
101	4.720	4.977	-0.257	5.093	-0.373
102	4.000	3.855	0.145	3.665	0.335
103	4.520	4.148	0.372	4.047	0.473
104	4.700	4.381	0.319	4.282	0.418
105	4.700	4.956	-0.256	5.122	-0.422
106	3.600	3.870	-0.270	4.112	-0.512
107	3.820	3.902	-0.082	4.088	-0.268
**Test**					
17	6.660	7.119	-0.459	7.092	-0.432
23	5.000	6.534	-1.534	6.619	-1.619
26	8.300	8.803	-0.503	8.389	-0.089
29	5.470	6.348	-0.878	6.391	-0.921
34	3.660	4.154	-0.494	4.349	-0.689
38	5.690	6.429	-0.739	6.351	-0.661
39	5.220	5.449	-0.229	5.571	-0.351
40	4.370	4.886	-0.516	5.021	-0.651
43	5.170	4.389	0.781	4.505	0.665
51	7.720	7.227	0.493	7.231	0.489
55	8.230	7.426	0.804	7.406	0.824
57	8.090	7.224	0.866	7.391	0.699
58	8.140	8.493	-0.353	8.495	-0.355
74	8.130	7.061	1.069	7.036	1.094
81	4.460	4.287	0.173	4.459	0.001
82	3.890	4.691	-0.801	4.712	-0.822
83	3.530	4.527	-0.997	4.777	-1.247
86	3.720	3.679	0.041	4.509	-0.789
87	3.600	4.587	-0.987	4.722	-1.122
89	3.600	3.677	-0.077	3.817	-0.217
90	3.960	3.979	-0.019	4.335	-0.375
91	3.450	3.658	-0.208	4.467	-1.017
98	3.600	5.162	-1.562	5.057	-1.457

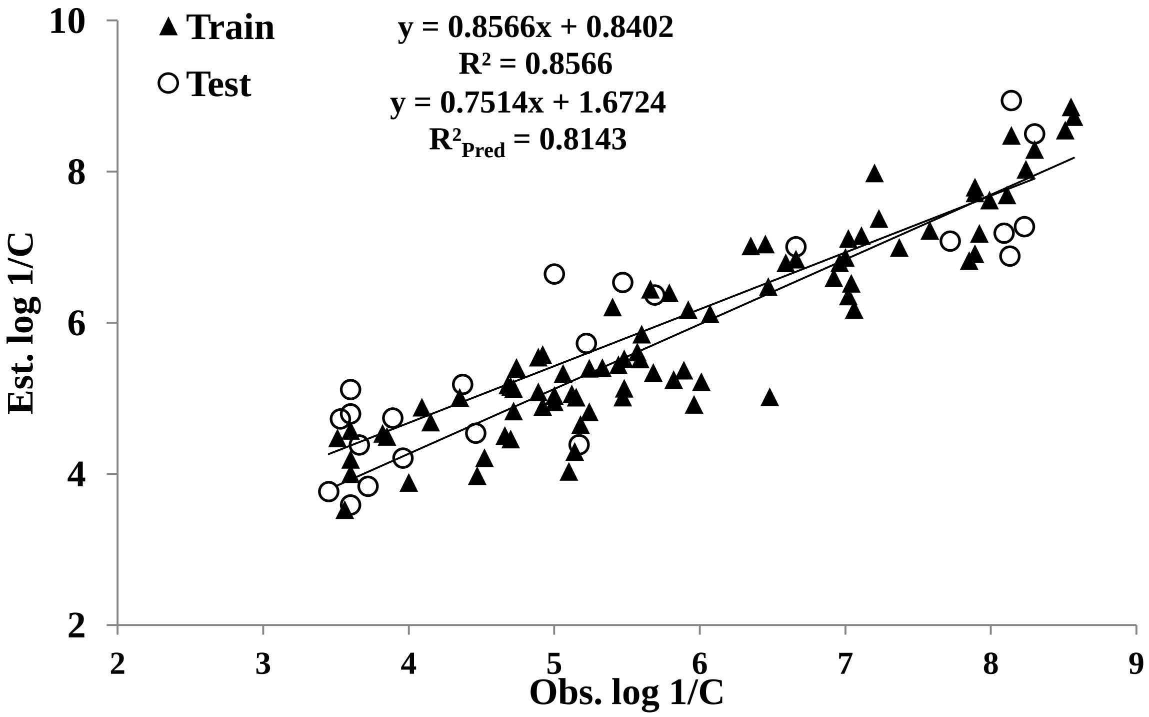

Figure S1 Correlation between observed and estimated values of log 1/C using eq. 4.

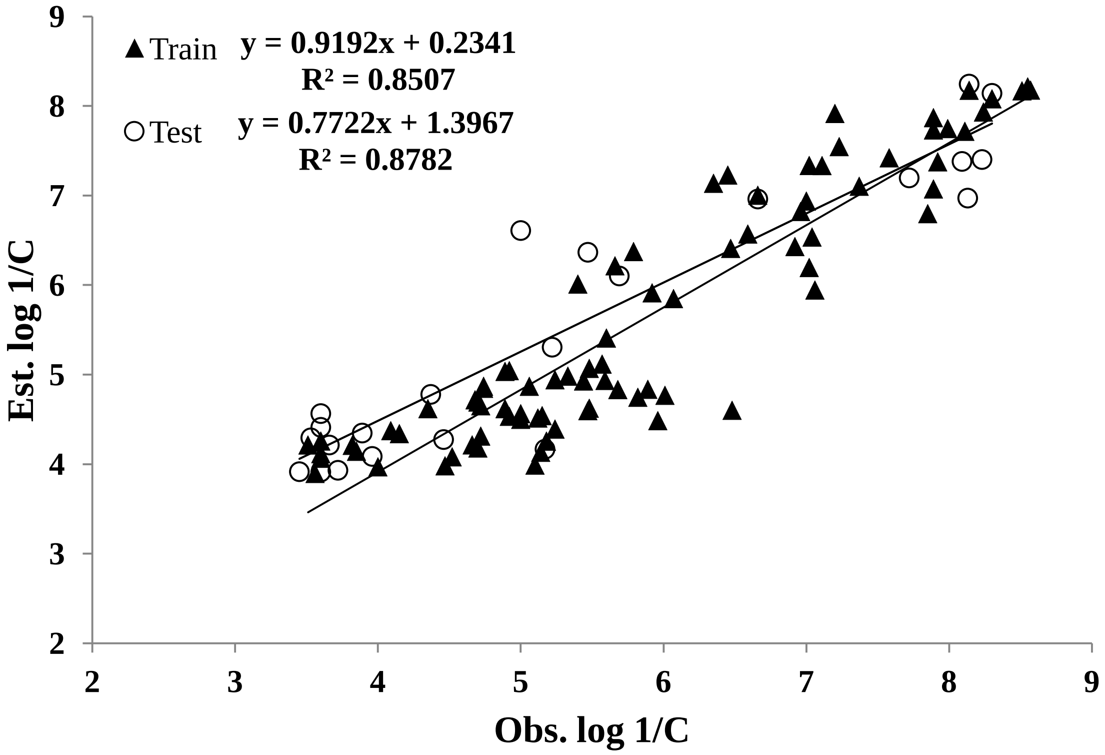

Figure S2. Correlation between observed and estimated values of log 1/C using eq. 4. (ANN)

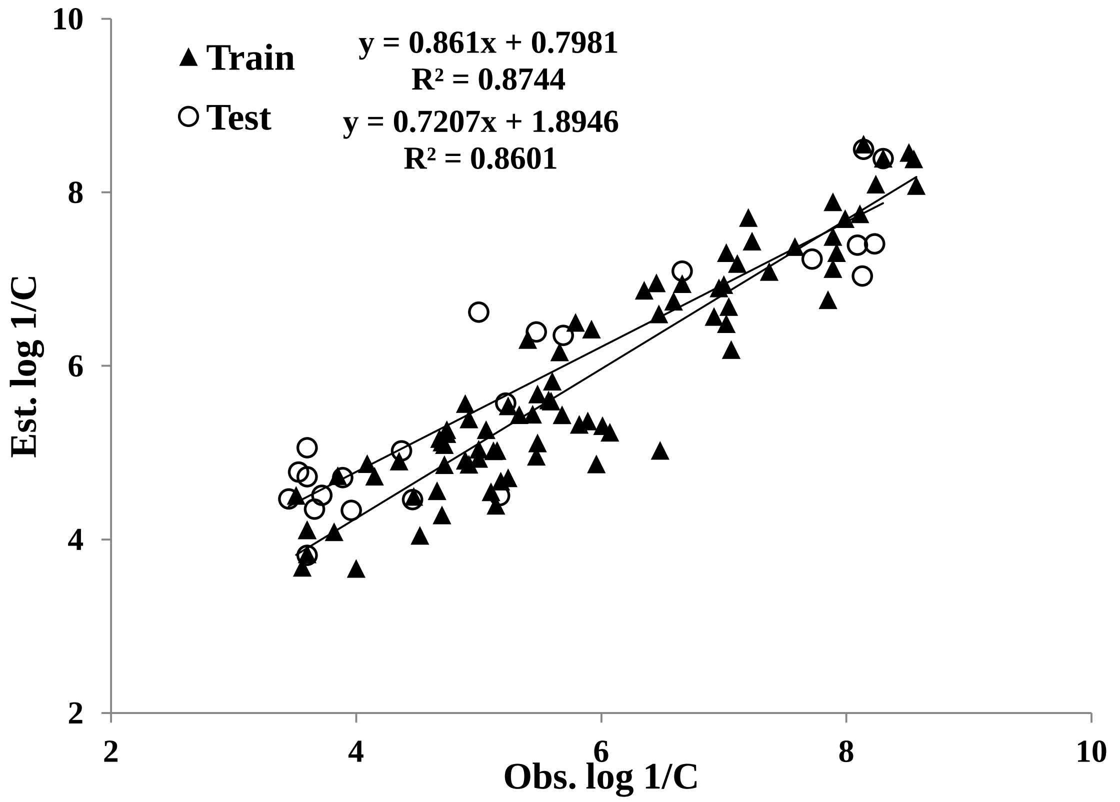

Figure S3. Correlation between observed and estimated values of log 1/C using eq. 4. (SVM)

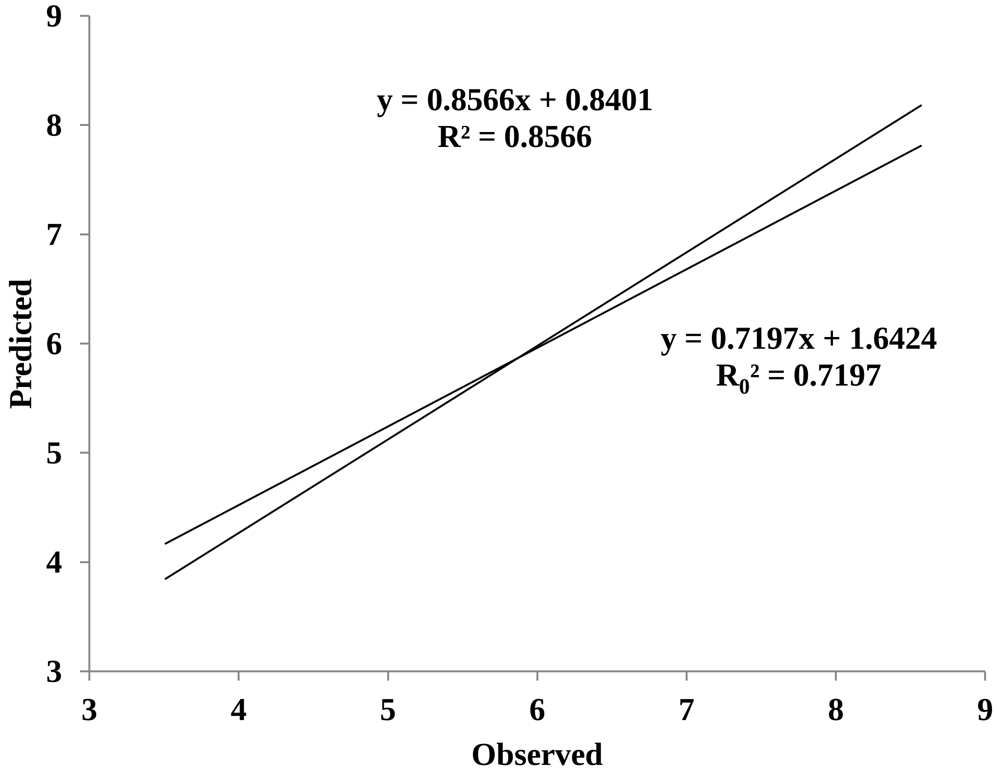

Figure S4 Randomization regression plot
